# Shared liability to pain, common mental disorders, and long-term work disability differs among women and men

**DOI:** 10.1097/j.pain.0000000000001787

**Published:** 2020-01-16

**Authors:** Jurgita Narusyte, Annina Ropponen, Ellenor Mittendorfer-Rutz, Pia Svedberg

**Affiliations:** a Division of Insurance Medicine, Department of Clinical Neuroscience, Karolinska Institutet, Stockholm, Sweden; b Finnish Institute of Occupational Health, Helsinki, Finland

**Keywords:** Work disability, Pain, Common mental disorders, Twin

## Abstract

Knowledge of factors involved in the associations between pain, common mental disorders, and future work incapacity is still scarce. The aim of this study was to examine the overlap between genetic and environmental factors contributing to depression/anxiety, pain, and future long-term sickness absence (SA) and disability pension (DP) among women and men. The study sample included 47,995 twins born in Sweden 1935 to 1985. Information on self-reported depression/anxiety and back, neck, and shoulder pain was obtained from surveys conducted 1998 to 2002 and 2004 to 2005. Data on long-term SA (>365 days) and DP due to mental and/or musculoskeletal disorders until 2013 were obtained from the National Social Insurance Agency. Shared genetic and environmental influences on depression/anxiety, pain, and SA/DP were estimated by applying structural equation modeling. The prevalence of depression/anxiety was 27% and 14% among women and men, for pain 24% and 19%, and for SA/DP due to mental and musculoskeletal diagnoses 7% and 4%, respectively. Multivariate biometric analyses revealed different patterns of covariation between the 3 phenotypes among women and men. For women, a latent-shared liability to all 3 phenotypes could be identified, mainly attributable to genetic factors (66%). For men, no shared underlying liability was observed. The variation in SA/DP was explained by genetic factors in common with depression/anxiety by 27% and in common with pain by 9%. Common mental disorders, pain, and SA/DP tend to covariate in different ways among women and men. The results may have clinical implications as strategies preventing SA/DP may be different among women and men.

## Abstract 2

Supplemental Digital Content is Available in the Text.

Etiology of covariation between common mental disorders, pain locations, and work disability tend to differ among women and men raising awareness for sex-specific pathways to work disability.

## 1. Introduction

Pain affects over 20% of the adult population and is the most common cause of long-term physical disability, and a substantial burden to both individuals and societies.^12,29^ Also, the increasing trends in common mental disorders (CMDs), such as depression and anxiety, are currently of great concern globally. Unipolar depressive disorder has, for example, been predicted to be the leading cause of loss of disability adjusted life years in high-income countries by 2030.^33^ The still rather limited knowledge of the factors involved in the progression from pain conditions and CMDs to work incapacity may be due to complexity, perhaps related to their common etiology, but also due to long-term effects of both groups of disorders.

Previous research suggests that pain and depression are strongly interlinked.^6,7,17^ In Europe, up to one-third of persons with chronic pain, most frequently back pain, have also been diagnosed with depression.^5,24^ However, whether mental disorders are a cause or a consequence of pain still remains unsolved. Anxiety may be a predictor of pain, whereas depression is claimed to be linked to the natural course of pain,^32,47^ potentially being a consequence of pain.^23,43^ Also a bidirectional link between pain and depression has been previously discussed and suggested to be attributable to shared underlying neurobiological and psychosocial factors.^10^

Two recent studies showed that the number of pain locations independently predicted disability pension (DP) due to musculoskeletal disorders and CMDs.^13,14^ Others have found that the ability to work and the degree of sickness absence (SA) in patients with chronic musculoskeletal pain was determined to a large extent by undiagnosed mental health comorbidities and not solely somatic complaints.^40^ Although additive effects^18^ and intercorrelated associations between CMDs and pain have been reported regarding the risk of DP,^41^ it is still unclear to what extent pain and CMDs share genetic and environmental risk factors with incident SA or DP.

Twin studies have reported moderate genetic influences on both musculoskeletal disorders and CMDs including low back pain (30%), osteoarthritis and disc degenerative disease (∼50%), depression (40%), and anxiety (20%).^4,15,16,20^ Genetic correlation between depressive symptoms and back pain was reported to be 0.48.^35^ Moderate genetic influences were also found on DP due to musculoskeletal disorders (35%-37%) and somewhat higher for DP due to CMDs.^11,38^ Interestingly, most genetic influences on incident DP due to CMDs do not seem to be explained by the genetic contributions to depression and anxiety disorders per se.^37^ Instead, the process leading to DP seems to be much more complex and influenced by factors other than those related to the disorder for which the DP was granted.

The aim was to investigate the etiology of the association between pain, CMDs, and future long-term work disability. Specifically, we investigated whether a shared liability could be identified between all 3 phenotypes among women and men and, if so, to what extent the shared liability is attributable to genetic and environmental factors.

## 2. Material and methods

### 2.1. Participants

This prospective study included all the twins identified in the Swedish Twin Registry (STR) born between 1925 and 1985, who are included in the Swedish Twin project of Disability pension and Sickness absence (STODS).^46^

Depending on the year of birth, twin individuals were either invited to participate in the Screening Across the Lifespan Twin Study, (SALT, birth cohort 1925-1958) in 1998 to 2003 or the Study of Twin Adults: Genes and Environment (STAGE, birth cohort 1959-1985) in 2004 to 2005. Both surveys included extensive batteries of questions regarding health, lifestyle, and sociodemographic characteristics.^27,28^ In total, 50,931 twin individuals responded.

Data on DP and SA during follow-up years 1998 to 2013 were obtained and linked to twins through registers at the Swedish Social Insurance Agency (SA, DP, and *ICD-10* diagnoses).^25,30^ Data on old-age retirement and year of emigration were obtained from the database LISA, Statistics Sweden, for years 1990 to 2012.^31^

The individuals included in the study were at risk for SA and DP, that is, younger than 65 years, not on long-term (>365 days) SA or DP, not on old-age pension, and with no self-reported cancer, rheumatoid arthritis, or ankylosing spondylitis at the baseline (ie, response data to either SALT or STAGE).

The final study sample included 47,995 twin individuals born 1935 to 1985, comprising 8715 monozygotic (MZ), 6981 same-sex dizygotic (DZ), and 7430 opposite-sexed dizygotic (DZ-OS) complete twin pairs.

### 2.2. Pain

The presence of pain was measured at baseline, that is, at the time of the SALT or the STAGE survey, and was based on the response to the survey questions: “Do you have or have you had back pain/neck pain/shoulder pain?” A binary variable *Pain* was created with value “0” if pain at none or one location was present and value “1” if individual experienced pain in at least 2 of the locations.

### 2.3. Common mental disorder

The presence of CMD was measured at baseline and included major depression (MD) and generalized anxiety disorder (GAD). In SALT, the presence of these was assessed using the computerized Composite International Diagnostic Interview-Short Form (CIDI-SF), adapted from its original design for 12-month prevalence of DSM-IV disorders.^21^ The assessment of MD and GAD in SALT is described in more detail elsewhere.^19,20^ Twins were considered to have a history of MD if they met criteria for MD or reported on present or previous use of antidepressant medication.^20^ In STAGE, the presence of MD was assessed using the Structured Clinical Interview for DSM-IV Disorders (SCID).^9^ Criteria A, C, and E had to be fulfilled for the participant to be classified as having a positive history of MD. In both SALT and STAGE, the individuals were considered as positive for a history of GAD if they reported on excess worry or anxiety that had lasted for at least 6 months (DSM-IV criterion A) and at least 3 of 5 symptoms (except “difficulty concentrating or mind going blank”) that were associated with worry and anxiety and had lasted for at least 6 months (criterion C).

For the purpose of this study, a binary variable CMD was created with a value “0” if no MD or GAD were reported and “1” in presence of at least one of these diagnoses.

### 2.4. Long-term work disability

All people in Sweden above the age of 16, with an income from work or unemployment benefits, can receive sickness benefits paid by the Social Insurance Agency when disease or injury have caused reduced work capacity. Employees get sick pay from their employers during the first 14 days after a qualifying day (usually more qualifying days for self-employed) without benefits. After 7 days of self-certification, a physician certificate is required. All people who due to disease or injury have a permanently impaired work capacity can be granted DP. The data used in this project include SA and DP benefits paid by the National Social Insurance Agency.^25,30^

A binary variable SA/DP for long-term work disability was created and included all new DP and >365-day SA cases due to mental (*ICD-10*: F00-F99) or musculoskeletal diagnoses (*ICD-10*: M00-M99). The variable SA/DP gained values “0” if no DP or SA and “1” if long-term SA or DP due to mental or musculoskeletal diagnoses had occurred during follow-up.

### 2.5. Statistical analyses

Descriptive statistics of the sample including prevalence rates of Pain and CMD at the baseline as well as prevalence of SA/DP during the follow-up were calculated for women and men. The first estimates of genetic and environmental influences on the phenotypes of interest (ie, Pain, CMD, and SA/DP) were obtained by comparing within-pair similarity in each twin zygosity group. In the twin method, we assume that MZ twins share 100%, and DZ twins share approximately 50% of their segregating genes. Thus, higher within-pair similarity among MZ than DZ twins would suggest that genetic factors are of importance, whereas comparable within-pair similarity between the MZ and DZ twins would suggest shared environmental (early family environment) influences. Within-pair similarity was measured by calculating intraclass tetrachoric correlation for MZ and DZ twins separately. The initial estimates of the genetic and environmental influences on the covariance between the phenotypes (ie, Pain, CMD, and SA/DP) among MZ and DZ twins were obtained by calculating cross-twin cross-trait tetrachoric correlations.

### 2.6. Biometric analyses

First, univariate models were fit to estimate the relative contributions of additive genetics (A), shared environment (C) or dominant genetic effects (D), and unique environmental effects (E) to the variance in Pain, CMD, and SA/DP. To test whether the estimates were different among women and men, a model was fitted where the genetic and environmental effects were different among women and men and then compared with the model when the estimates were constrained to be equal between the sexes.

Second, 3 multivariate genetic models were applied to decompose the covariance between Pain, CMD, and SA/DP into genetic and environmental components. The purpose of the multivariate genetic analyses was to examine to what extent the total variation in SA/DP was explained by factors contributing to pain and CMD. A trivariate Cholesky decomposition model (1) was fitted to the data, followed by Independent Pathway (2) as well as Common Pathway (3) models.^39^ In the Cholesky model, the first genetic or environmental factor loads on all 3 phenotypes (ie, CMD, Pain, and SA/DP), the second factor loads on all except the first, and the third factor only loads on the third phenotype. In the Independent Pathway model, genetic and environmental factors are of 2 types: common and phenotype-specific. Common genetic and environmental factors load on all 3 phenotypes, whereas phenotype-specific loads on each phenotype. In the Common Pathway model, common genetic and environmental influences are mediated through a shared latent factor that represents a common liability to Pain, CMD, and SA/DP. In addition to a shared latent factor, also genetic and environmental influences specific to each phenotype are estimated. The Common Pathway model has fewer parameters as compared to the Independent Pathway model and is more parsimonious. Akaike's Information Criterion and Bayesian Information Criterion were used to evaluate the model best fitting our data, where the lower Akaike's Information Criterion and Bayesian Information Criterion values indicate a model of a better fit and greater parsimony (ie, fewer parameters).

Genetic (*r*_g_) and environmental (*r*_e_) correlations between CMD and SA/DP as well as Pain and SA/DP were estimated. A genetic correlation indicates the extent to which genetic effects on SA/DP overlap with genetic effects on Pain or CMD. Similarly, for shared and nonshared environmental correlations, these indicate to what extent environmental effects on SA/DP overlap with environmental effects on Pain or CMD. In addition, we calculated the extent to which the covariance between the 3 phenotypes could be explained by genetic or environmental effects contributing to Pain and/or CMDs.

Data analyses were performed by statistical software SAS V 9.4 and Mx.

## 3. Results

Descriptive statistics for the total study sample as well as separately for participants in SALT and STAGE are presented in Table 1.

**Table 1:**
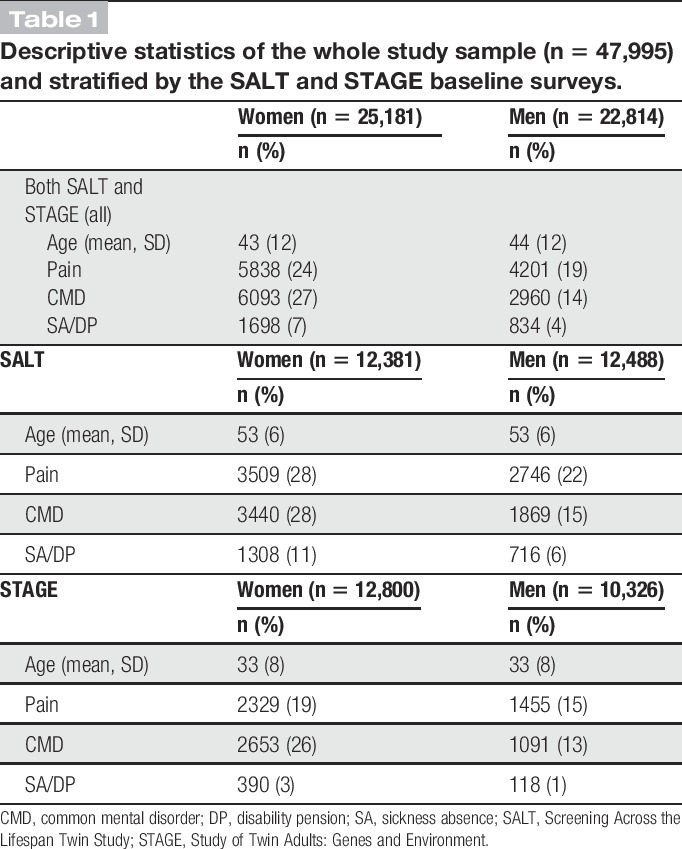
Descriptive statistics of the whole study sample (n = 47,995) and stratified by the SALT and STAGE baseline surveys.

The phenotypic correlation between CMD and Pain was 0.19, between CMD and SA/DP was 0.24, and between Pain and SA/DP was 0.27. Intraclass and cross-twin cross-trait correlations are presented in Table 2. Intraclass correlations among MZ twins were approximately twice the correlations among DZ twins for all 3 phenotypes, suggesting that genetic influences may be of importance. Intraclass correlations among opposite-sexed DZ twins were lower as compared to same-sexed DZ twins for CMD and SA/DP, suggesting that the estimates may be different among women and men.

**Table 2:**
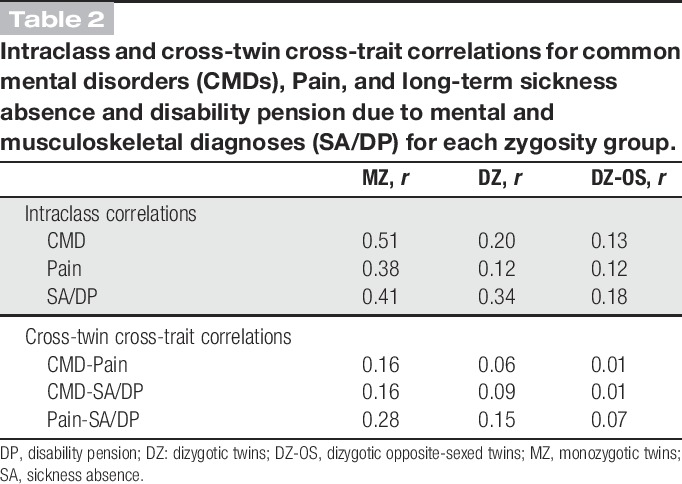
Intraclass and cross-twin cross-trait correlations for common mental disorders (CMDs), Pain, and long-term sickness absence and disability pension due to mental and musculoskeletal diagnoses (SA/DP) for each zygosity group.

Cross-twin cross-trait correlations were higher among MZ twins as compared to DZ twins, suggesting that genetic influences were of importance for the covariance between the phenotypes. The correlations among opposite-sexed DZ twins were slightly lower than compared with same-sexed DZ twins, suggesting that sex differences may be present.

In Table 3, the results of the best-fitting univariate models for CMD, Pain, and SA/DP are shown (model comparison results not shown). The best-fitting model was identified by running a series of models allowing for quantitative sex differences. For CMD, the best-fitting model was ADE with different estimates for women and men. For Pain, the ADE model was the best-fitting for women, whereas AE model was best-fitting for men. For SA/DP, the best-fitting model was AE with different estimates for women and men. Thus, the following multivariate analyses were conducted separately for women and men.

**Table 3:**
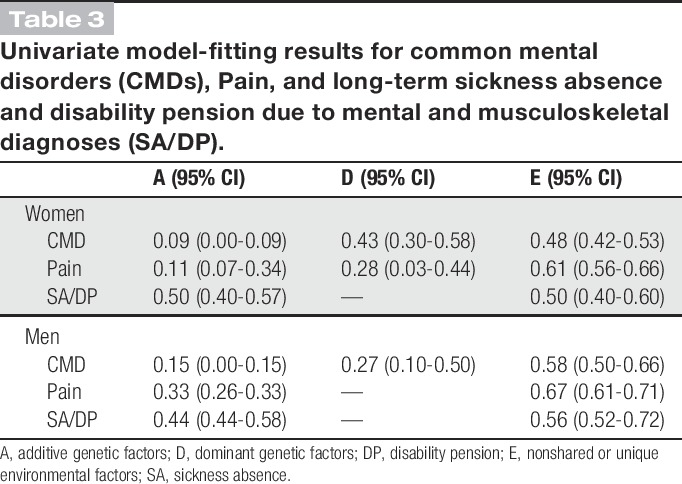
Univariate model-fitting results for common mental disorders (CMDs), Pain, and long-term sickness absence and disability pension due to mental and musculoskeletal diagnoses (SA/DP).

**Figure 1.**
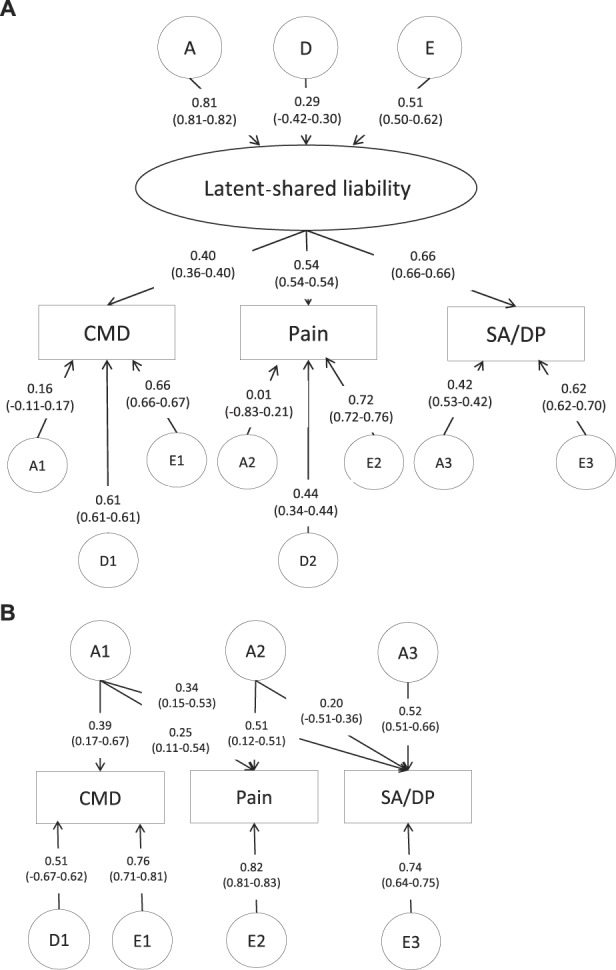
Standardized estimates for the common pathway model for women (A) and Cholesky decomposition model for men (B). (A) Women (A: common additive genetic factors, D: common dominant genetic factors, E: common nonshared or unique environmental factors, A1–A3: specific additive genetic factors, D1–D2: specific dominant genetic factors, E1–E3 specific unique environmental factors). (B) Men (A1–A3: additive genetic factors, D1: dominant genetic factors, E1–E3: nonshared or unique environmental factors).

The results of multivariate genetic analyses are presented in Table 4. In the analyses, the dominant genetic components for SA/DP were dropped after the results from the univariate analyses. For women, the best-fitting and most parsimonious model was a common pathway model. The standardized estimates of the model are presented in Figure 1A. Additive genetic effects contributed to the variance of the common latent factor, called latent-shared liability, by 66% (0.81^2^), dominant genetic effects by 8% (0.29^2^, nonsignificant) and nonshared environmental effects by 26% (0.51^2^) (Fig. 1A). The breakdown of total variance into common and specific components for each of the phenotypes is presented in Table 5. Almost half of the total variance of CMD, Pain, and SA/DP was explained by specific nonshared environmental effects. Variance in common to CMD, Pain, and SA/DP was mainly attributable to common additive genetic factors by 10% (0.81^2^ × 0.40^2^), 19% (0.81^2^ × 0.54^2^), and 29% (0.81^2^ × 0.66^2^), respectively.

**Table 4:**
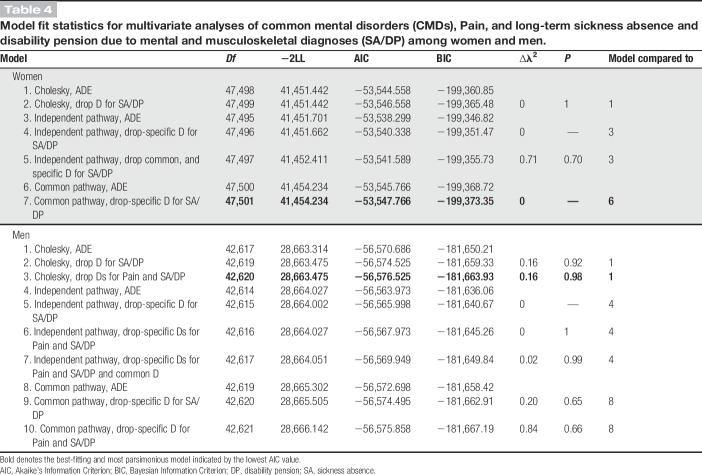
Model fit statistics for multivariate analyses of common mental disorders (CMDs), Pain, and long-term sickness absence and disability pension due to mental and musculoskeletal diagnoses (SA/DP) among women and men.

**Table 5:**
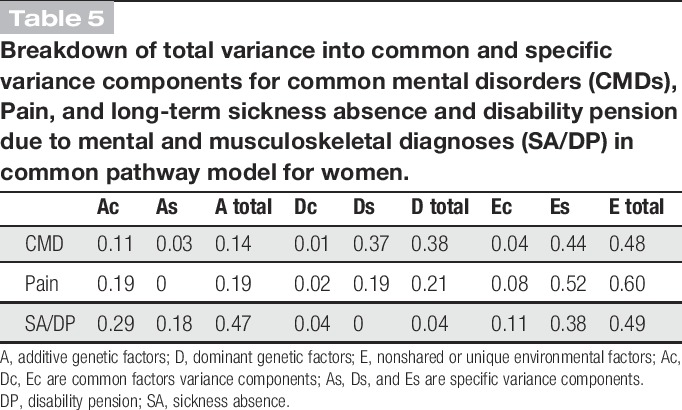
Breakdown of total variance into common and specific variance components for common mental disorders (CMDs), Pain, and long-term sickness absence and disability pension due to mental and musculoskeletal diagnoses (SA/DP) in common pathway model for women.

For men, the Cholesky decomposition model explained the data in the best and most parsimonious way. The standardized estimates of the model are presented in Figure 1B. The covariation between all 3 phenotypes seemed to be mainly due to common additive genetic factors. The total genetic variance of Pain was explained by 19% (0.25^2^/0.25^2^ + 0.51^2^) of genetic factors in common with CMD and 81% by genetic factors unique to Pain. The total genetic variance of SA/DP was explained by genetic factors contributing to CMD by 27% (0.34^2^/0.34^2^ × 0.20^2^ + 0.52^2^), genetic factors contributing to Pain by 9% (0.20^2^/0.34^2^ × 0.20^2^ + 0.52^2^), and genetic factors that were unique to SA/DP by 63% (0.52^2^/0.34^2^ × 0.20^2^ + 0.52^2^) (Fig. 1B). Nonshared environmental factors were mostly unique to each of the phenotypes (CMD, Pain, and SA/DP). For men, the additive genetic correlation (*r*_g_) between CMD and SA/DP was 0.68 and 0.67 between Pain and SA/DP.

Multivariate genetic analyses were also performed for each of the cohorts, SALT and STAGE, separately. The results followed the same pattern as in the analyses of both cohorts together. That is, common pathway model and Cholesky decomposition model were the best-fitting and most parsimonious models among women, respectively, men in both SALT and STAGE (Tables S1 and S2, available at http://links.lww.com/PAIN/A935).

## 4. Discussion

In this prospective population-based study with almost 48,000 twin individuals, we investigated the common etiology of the covariation between CMDs, pain, and future work disability in terms of long-term SA and DP among women and men. For women, we found the covariance between all 3 phenotypes could be explained by a latent shared liability. For men, genetic factors contributing to the variation in CMD were also contributing to SA/DP to a greater extent than genetic factors contributing to pain.

Our finding of different etiological patterns across sexes regarding covariation between CMDs, pain, and work disability is in line with reports of different etiology between sexes for depression and pain.^3,20^ For example, higher heritability estimates among women than men were observed for MD (42% and 29%, respectively), while heritability for low back and neck pain seem to be higher among men than women,^8,20^ but studies are also consistent that women are at a larger risk for pain as well as experience higher pain sensitivity compared with men.^3^ Although research is scarce regarding sex differences in heritability for SA and DP, differences in SA and DP prevalence between women and men is a well-acknowledged fact.^1,38,45^

A latent-shared liability underlying the covariation among pain, CMDs, and SA/DP was suggested among women but not men. One explanation to this finding may be related to sex differences in comorbidity between CMDs and pain.^34^ The comorbidity between pain and depression is well documented, where prevalence of comorbid pain and depression is higher than the prevalence of each condition alone^2^ and higher among women compared with men.^36^ Higher levels of comorbidity among women than men may be related to greater health impairment and work disability and thus have greater impact on the process of SA or DP grant. Of important note is that the comorbidity between pain and depression is complex and suggested to be reciprocal as well as may exacerbate each other as suggested by preclinical and clinical studies.^26^

The variance of a common latent factor among women was mainly explained by additive genetic effects. That is, the covariation among CMDs, pain, and SA/DP seem to be mainly attributable to a shared genetic liability. A previous study has reported that shared genetic factors contributed significantly to the covariance between low back pain and depression.^42^ Also, another study has shown that genetic influences on CMDs explained 31% of the total variance in DP due to mental diagnoses among women.^37^ Genetic influences on the common latent factor can be attributable to molecular mechanisms behind comorbidity between pain and depression (eg, decreased availability in monoamine neurotransmitters such as serotonin or dopamine) or response to inflammatory factors.^44^ However, a common genetic liability behind all 3 phenotypes does not exclude prevention or intervention possibilities. Namely, most of the variance in each of the phenotypes was explained by specific nonshared environmental factors suggesting that different environmental factors are of importance, and that these could be approached in preventive strategies of work disability in health care.

### 4.1. Strengths and limitations

Strengths of this study include the large population-based sample size, no loss-to-follow regarding long-term SA and DP, and unique survey data. Because of the large high-quality sample size, we could study the patterns of covariation separately among women and men, which is rarely possible otherwise. Several limitations also need to be mentioned. First, the time-point of conducting SALT and STAGE surveys could differ for several years and may contribute to the measurement error term in CMD and pain. However, the analyses of each cohort showed similar results. Second, despite the large sample size, the number of SA/DP due to mental and musculoskeletal diagnoses was too low to analyze the data for each diagnosis group separately. Although CMDs and pain are frequently comorbid with several other physical conditions, the covariance between the pain, CMD, and SA/DP might have been more pronounced if SA/DP spells due to mental or musculoskeletal diagnoses were analyzed separately. Third, the number of SA/DP was much lower in STAGE as compared to SALT cohort. Respondents to STAGE were younger, and from previous research, it is well known that higher age is a risk factor for SA/DP.^1^ Hence, results should be interpreted with caution regarding younger age groups. Fourth, the phenotypes were measured in different ways, that is, using self-report, DSM-IV–based questions as well as register data. Although self-reported data may be of lower precision due to, for example, recall bias, there are no other means to measure the presence or level of pain among thousands of individuals.^22^ Finally, a broad definition was used to define presence of pain in this study, which possibly could lead to including low intensity or temporary pain conditions. Also, no clear distinction was made between chronic and acute pain conditions having different underlying mechanisms, which could not be considered in this study. Hence, the results of the study need to be replicated in future studies.

In conclusion, CMDs, pain, and future SA/DP tend to covariate in different ways among women and men. Although needed to be replicated in future studies, the results raise awareness for different strategies preventing SA/DP among women and men.

## Conflict of interest statement

The authors have no conflicts of interest to declare.

## Appendix A. Supplemental digital content

Supplemental digital content associated with this article can be found online at http://links.lww.com/PAIN/A935.

## Supplementary Material

SUPPLEMENTARY MATERIAL
