# Supplementary material for: Shared liability to pain, common mental disorders, and long-term work disability differs among women and men
Source: Pain. 2020 Jan 16;161(5):1005–11. doi: 10.1097/j.pain.0000000000001787 (PMC7170444; doi:10.1097/j.pain.0000000000001787)
Supplement: SUPPLEMENTARY MATERIAL [file jop-161-1005-s001.pdf]

Table S1. Model fit statistics for multivariate analyses of common mental disorders (CMD), Pain, and long-term sickness absence and disability pension due to mental and musculoskeletal diagnoses (SA/DP) among women and men in SALT cohort

Model	df	-2LL	AIC	BIC	$\Delta\lambda^2$	p	Model compared to
**Females**							
1. Cholesky, ADE	23191	23017.947	-23364.053	-87536.200			
2. Cholesky, drop D for SA/DP	23192	23017.947	-23366.053	-87540.471	0.00	0.99	1
3. IP, ADE	23188	23017.974	-23358.026	-87523.375			
4. IP, drop specific D for SA/DP	23189	23017.974	-23360.026	-87527.645	0	0.99	3
5. IP, drop common and specific D for SA/DP	23190	23019.015	-23360.985	-87531.396	1.04	0.59	3
6. CP, ADE	23193	23020.721	-23365.279	-87543.355			
**7. CP, drop specific D for SA/DP**	**23194**	**23020.721**	**-23367.279**	**-87547.626**	**0**	**0.99**	**6**
**Males**							
1. Cholesky, ADE	23142	17773.034	-28510.966	-89881.441			
2. Cholesky, drop D for SA/DP	23143	17773.034	-28512.966	-89885.709	0		1
**3. Cholesky, drop Ds for Pain and SA/DP**	**23145**	**17773.034**	**-28516.966**	**-89894.245**	**0**	**-**	**1**
4. IP, ADE	23139	17774.028	-28503.972	-89868.140			
5. IP, drop specific D for SA/DP	23140	17774.028	-28505.972	-89872.408	0	0.99	4
6. IP, drop specific Ds for Pain and SA/DP	23141	17774.028	-28507.972	-89876.676	0	-	4
7. IP, drop specific Ds for Pain and SA/DP and Common D	23142	17774.096	-28509.904	-89880.910	0.07	0.99	4
8. CP, ADE	23144	17774.946	-28513.054	-89889.021			
9. CP, drop specific D for SA/DP	23145	17775.020	-28514.980	-89893.251	0.08	0.79	8
10. CP, drop specific D for Pain and SA/DP	23146	17776.808	-28515.192	-89896.626	1.86	0.39	8

Note. Bold denotes the best-fitting and most parsimonious model indicated by the lowest AIC-value.

Table S2. Model fit statistics for multivariate analyses of common mental disorders (CMD), Pain, and long-term sickness absence and disability pension due to mental and musculoskeletal diagnoses (SA/DP) among women and men in STAGE cohort

Model	df	-2LL	AIC	BIC	$\Delta\lambda^2$	p	Model compared to
**Females**							
1. Cholesky, ADE	24288	17900.368	-30675.632	-95553.638			
2. Cholesky, drop D for SA/DP	24289	17900.368	-30677.632	-95557.941	0	-	1
3. IP, ADE	24285	17900.494	-30669.506	-95540.667			
4. IP, drop specific D for SA/DP	24286	17900.493	-30671.507	-95544.970	0	-	3
5. IP, drop common and specific D for SA/DP	24287	17901.215	-30672.785	-95548.912	0.72	0.70	3
6. CP, ADE	24290	17901.823	-30678.177	-95561.516			
**7. CP, drop specific D for SA/DP**	**24291**	**17901.823**	**-30680.177**	**-95565.819**	**0**	**-**	**6**
**Males**							
1. Cholesky, ADE	19456	10499.576	-28412.424	-77161.626			
2. Cholesky, drop D for SA/DP	19457	10499.576	-28414.424	-77165.861	0	-	1
**3. Cholesky, drop Ds for Pain and SA/DP**	**19459**	**10499.576**	**-28418.424**	**-77174.333**	**0**	**1**	**1**
4. IP, ADE	19453	10500.274	-28405.726	-77148.569			
5. IP, drop specific D for SA/DP	19454	10500.328	-28407.672	-77152.778	0.05	0.82	4
6. IP, drop specific Ds for Pain and SA/DP	19455	10500.328	-28409.672	-77157.014	0.05	0.97	4
7. IP, drop specific Ds for Pain and SA/DP and Common D	19456	10500.328	-28411.672	-77161.249	0.05	0.99	4
8. CP, ADE	19458	10504.416	-28411.584	-77167.677			
9. CP, drop specific D for SA/DP	19459	10504.476	-28413.524	-77171.883	0.06	0.81	8
10. CP, drop specific D for	19460	10504.439	-28415.561	-77176.137	0.06	0.97	8

Pain and SA/DP

Note. Bold denotes the best-fitting and most parsimonious model indicated by the lowest AIC-value.
